# Gas in the Stomach; Its Causation and Treatment

**Published:** 1911-02-25

**Authors:** Frank Kennedy Cahill


### Hospital Clinics.

### GAS IN THE STOMACH; ITS CAUSATION AND TREATMENT.

By FRANK KENNEDY CAHILL, F.R.C.S., D.P.H.

A Paper read in the Section of Medicine in the Royal Academy of Medicine in Ireland.

To quote the words of Professor Reed, "Such an important and obtrusive symptom as 'flatulency' needs to be elucidated and traced to its possible causes in language easily intelligible to every practitioner of Medicine."

At the outset I should like to define with a certain exactitude some terms employed—more especially "fermentation" and "putrefaction." It is unfortunate that these terms are frequently used as synonyms. *Clinically* speaking, it seems reasonable to suggest that the term "fermentation" should be used only to designate decomposition of carbohydrates.

There are four kinds of this sort of fermentation found in human stomachs: (*a*) lactic acid, (*b*) butyric acid, (*c*) acetic acid, (*d*) yeast fermentation. These form various products which may recombine among one another, the most characteristic products being (1) alcohol, (2) methane, (3) ethylene, besides the acids mentioned. The term "putrefaction" should be, I think, applied only to the decomposition of albuminous or proteid bodies.

While a few of the same products arise as in fermentation, the main end-products of "putrefaction" may be arranged in three groups: I. (1) Ammonia, (2) nitrogen, (3) carbon dioxide, (4) sulphuretted hydrogen, (5) methylmercaptan, (6) cystin, &c. II. Series of amido acids. III. Aromatic derivatives of benzol, phenol, cresol, phenol-acetic acid, indol, skatol, tyrosin. We may contrast "fermentation" and "putrefaction," at least so far as they occur in the alimentary canal, roughly as follows:—

| Fermentation | Putrefaction |
|---|---|
| 1. Involves carbohydrates | 1. Involves fats and proteids |
| 2. Produces acids mainly | 2. Produces alkalies mainly |
| 3. Numerous bacteria, especially long threads and yeasts or fungi | 3. Colon bacillus mainly |
| 4. Mainly in stomach or upper portion of alimentary canal | 4. Mainly lower bowel and colon |

Having discussed these terms, I now proceed to enumerate the various causal factors in the production of gastric gas.

(1) Air swallowing, (2) chemic gastric respiration, (3) mechanic gastric respiration, (4) inorganic production of $CO_2$, (5) organic production of $CO_2$ and associated gases, (6) spasm of cardia or pressure from without.

(1) *Air Swallowing.*—Deglutition always carries more or less air into the stomach. Normally the amount is insignificant, but, owing to rapid eating or drinking, gulping down large quantities of food or drink in a single bolus, and undoubtedly to peculiarities in the shape and mode of closure of the fauces and pharynx,* so much air may be swallowed as to cause an excess of endogastric tension during and immediately after a meal. This tension is usually relieved by belching. The differential diagnosis depends upon (*a*) the time of belching, (*b*) the lack of odour of putrefaction or fermentation, (*c*) the absense of the neurotic element, (*d*) the normality of the gastric contents. Treatment depends rather on attention to etiquette than hygiene or medication. Though one has never been able actually to demonstrate an anomaly of the fauces or pharynx, yet the occurrence of this trouble in persons whose habits of swallowing are good suggests such a condition, and one is at a loss to suggest an appropriate means of correction.

(2) *Chemic Gastric Respiration.*—Benedict and Reed deny the existence of chemic gastric respiration. According to the former, "All active cells absorb oxygen and give off $CO_2$, not to mention other katabolic products; but it is absurd to suppose that the thick glandular lining of the stomach can be compared to the diaphanous, squamous lining of the pulmonary vesicles. Moreover, respiration is an exchange, not a production, and it is impossible that any appreciable quantity of gas can be eliminated into the stomach by this means." The latter says: "I have not been able to convince myself that gases are secreted directly by the cells of the stomach, as has been claimed by some authors." On the other hand, Hutchison quotes Evans, who brought forward evidence, derived from a study of the gas contained in the swim-bladder of fish, in favour of the view that some of the nitrogen may be derived from the blood. Evans (*British Medical Journal*, 1897, page 649) bases his view on the result of two analyses. As this method of gas production is common to all stomachs it cannot be a very potent factor leading to flatulency of a distressing nature. On the other hand, if we do not accept the view that gases are transfused from or secreted by the blood we are at a loss to account for the sudden appearance of gaseous distension after abdominal operations, in intestinal obstruction, &c.

(3) *Mechanic Gastric Respiration.*—In hiccough there is necessarily a tendency for air to enter the œsophagus and stomach. Since the exciting cause is usually too rapid swallowing or swallowing of dry foods, it seems that hiccough is essentially conserva-

* The writer, since this paper was written, has observed two cases of "air swallowers," who presented a remarkable enlargement of the bodies of cervical vertebræ which protruded forward into the pharynx, so that when the examining finger passed the soft palate it immediately encountered this prominence. In both these cases there was no other discoverable cause for their "wind." Both, however, were neurotics and allowed one to sweep the finger around the pharynx without their experiencing any discomfort.

tive—nature's relief of the œsophageal spasm by dilating this tube with air, and, so to speak, endeavouring to blow through into the stomach any obstruction that may be present. In dilated or relaxed conditions of the œsophagus and cardia there may be then a suction into and expulsion from the stomach of air, as well as pulmonary inspiration and expiration. When, therefore, in neurotic patients, without evidence of gastric fermentation, and with somewhat conspicuous respiratory movements, there is repeated noisy eructation of gas, this condition should be suspected. There exists a disturbance of innervation, usually a laryngeal spasm, and, after the primary œsophageal spasm has subsided, a relaxation of the œsophagus. As to treatment, forced respirations, anti-spasmodic drugs, and the various devices suggested for the relief of hiccough, may be employed, even when this system is absent. Personally, I have found "lavage" quite satisfactory. It is possible that the cases of gastric gas reported as due to gastric respiration in the chemic sense (2) are mainly mechanic or due to the inorganic production of $CO_2$, the next condition to be described.

(4) *Inorganic Production of* $CO_2$.—Benedict lays stress on this mode of production, considering that there is present a super-secretion of gastric, pancreatic, and intestinal juices and bile with more or less patency of the pylorus, and that the meeting of hydrochloric acid with alkaline carbonates produces carbon dioxide. He contends that there are occasional cases in which enormous quantities of nearly odourless gas, shown by simple tests to consist largely of $CO_2$, are given off by belching, and whose origin cannot be accounted for by any of the theories hitherto put forward. (We must remember that this writer denies the existence of chemic gastric respiration.) The patients are neurotic, the waterbrash, or vomited matter or wash water, is nearly clear, and contains nothing but a little mucus and the usual slight debris of a jejune stomach, except that, occasionally, bile pigment may be seen. Effervescence is heard in the region of the pylorus. Especially do these cases fail to give evidence of fermentation and to respond to antiseptics unless the antiseptics are distinctively sedative. Our knowledge of the amounts of these secretions and of their percentage of acid and alkali shows that a very considerable amount of gas might be produced. In these cases the failure of antiseptics will often suggest the proper diagnosis, or, at least, will lead to the employment of sedatives. Bismuth is indicated locally, the coal tar and mydriatic anodynes by the mouth.

(5) *Organic Production of* $CO_2$ *and Associated Gases*.—With all the favouring conditions present it is a wonder that the gastric bacteria and fungi do not invariably cause a notable production of the gases of putrefaction and fermentation, especially as there is so often superadded a supply of germs from septic stumps and nasal cavities. The lack of such gas production is ascribed to (*a*) the comparative shortness of gastric digestion, (*b*) the removal of fermentable and putrescible material by digestion and absorption, (*c*) the antiseptic action of hydrochloric acid. Most authorities consider the last as relatively unimportant on account of the dilution of the acid; but it must be of considerable importance, as the other circumstances are merely negative. It must be remembered, moreover, that the standard of the bacteriologist is a lethal one, that of the physiologist and the therapeutist merely inhibition of the vegetable cell. I take it, then, that gastric putrefaction may be assigned to two sets of causes, disturbance of intestinal peristalsis, including cancer, and the various types of ulceration. The treatment of the underlying condition, often involving surgical interference, is beyond the scope of this paper. The milder measures which are usually ample in the treatment of fermentation cannot be relied upon to cleanse a stomach in which proteolytic bacteria are at work. Various antiseptics have been used, but I think a 4 per cent. solution of borax and sod. bicarb. will be found efficient. While it is impossible to disinfect by the mouth a foul stomach full of chyme, it is perfectly possible to keep a jejune one relatively aseptic by administering salacetol, or some similar antiseptic, with moderate amounts of water. Hence the value of administering nutrient enemata and cutting off all nourishment by the mouth.

Again, gastric fermentation is often considered as amylaceous dyspepsia; starches are withheld as far as possible, and diastase is given to ensure their prompt digestion. While it is true that "ptyalin" digests cooked starches into maltose, and that small quantities of "invertin" are secreted by the stomach, to convert this maltose into glucose, the amount of starch digestion that normally takes place in the stomach is insignificant. When much gas is formed in the stomach from carbohydrates the reason is usually that there is a failure of normal antisepsis due to hypochlorhydria.

In estimating the "acidity" due to HCl one may be deceived by testing the "filtrate" after a test-breakfast, as a weak stomach may react normally under the light burden of that meal, though it may not produce sufficient HCl under normal diet; just as a weak spring may rise as high as a strong one under a small load.

While gas production from proteids requires mechanic cleansing of the stomach and physiologic rest, carbohydrate fermentation may be treated by antiseptics. Usually, there are also indications to increase the amount of HCl both by its administration as such, by increasing the chlorides, by giving sodium chloride in excess at the meals, and stimulating the gastric glands by giving strychnine. Charcoal, if given in large doses, is of some value in fixing gas, but it will not prevent gas formation.

(6) When, through spasm of the cardia or pressure from without, gas, however produced, is not freely eructated, it must either pass through the pylorus or produce characteristic symptoms from distension of the stomach. The cases in which gas is produced by a mingling of gastric and alkaline intestinal juices cannot lead to distension of the stomach alone, but spasm, kinking, or other obstruction below the pylorus may confine the gas of the stomach and part of the upper small intestine. Most cases, however, seem to be due to organic formation of gas, and mainly to fermentation of carbohydrates. Mild forms are best treated by the administration of hot carminative drinks, change of posture, &c. Such cases are very common ; they are especially frequent at night, perhaps because some posture during sleep favours pressure of the liver on the pylorus, duodenum, or upper coils of the jejunum ; perhaps because lessened peristalsis allows an accumulation of solid and liquid intestinal contents, so that a trap is formed in the intestine. Whether the closure above is entirely spastic—and one would scarcely consider sleep to favour the development of spasm—or due to the pressure of the liver on the œsophagus, it is difficult to say ; but there is no question that gastric distension is favoured by lying on the left side.

"Nightmare" is frequently due to this cause, and one can only decide by the state of the pulse and the heart sounds in any particular case whether the sense of cardiac interference is mainly genuine or due to the "nightmare." Most of these cases are relieved spontaneously or by domestic treatment ; and apart from the passage of the stomach-tube to relieve gas-pressure the treatment is prophylactic, to prevent the formation of gas, a subject which has been dealt with in the course of this paper. Having passed, in somewhat sketchy review, the various theories of gas formation in the stomach, I would, in conclusion, enter a strong plea for the more careful investigation of these cases, so often recurring in general practice, and instead of routine mixtures for "wind" so often prescribed I would substitute a minute and searching inquiry into primary causes—their removal resulting in credit to ourselves and service to our patients.

